# The SIGN nail for knee fusion: technique and clinical results

**DOI:** 10.1051/sicotj/2015038

**Published:** 2016-02-05

**Authors:** Duane Ray Anderson, Lucas Aaron Anderson, Justin M. Haller, Abebe Chala Feyissa

**Affiliations:** 1 Soddo Christian Hospital Soddo Ethiopia; 2 University of Utah Department of Orthopaedics Salt Lake City Utah USA

**Keywords:** Knee fusion, Intramedullary nail, SIGN nails

## Abstract

*Purpose*: Evaluate the efficacy of using the SIGN nail for instrumented knee fusion.

*Methods*: Six consecutive patients (seven knees, three males) with an average age of 30.5 years (range, 18–50 years) underwent a knee arthrodesis with SIGN nail (mean follow-up 10.7 months; range, 8–14 months). Diagnoses included tuberculosis (two knees), congenital knee dislocation in two knees (one patient), bacterial septic arthritis (one knee), malunited spontaneous fusion (one knee), and severe gout with 90° flexion contracture (one knee). The nail was inserted through an anteromedial entry point on the femur and full weightbearing was permitted immediately.

*Results*: All knees had clinical and radiographic evidence of fusion at final follow-up and none required further surgery. Four of six patients ambulated without assistive device, and all patients reported improved overall physical function. There were no post-operative complications.

*Conclusion*: The technique described utilizing the SIGN nail is both safe and effective for knee arthrodesis and useful for austere environments with limited fluoroscopy and implant options.

## Introduction

While knee arthrodesis is an uncommon procedure in the modern era of knee arthroplasty, it remains useful when other surgical procedures are contraindicated. The most common indication for knee arthrodesis currently is failed total knee arthroplasty, though serious bacterial infections with bone destruction, advanced tuberculosis, polio with flexion contracture, and severe contractures are also indications for knee arthrodesis [1].

There are several implants that have been used to instrument a knee arthrodesis. Sophisticated two-part intramedullary nails are used in the developed world for knee fusion [2]. The SIGN nail is a simple, stable, and inexpensive implant that can be used for knee fusion and is available in many institutions in developing countries [3]. Additionally, SIGN intramedullary nails do not require fluoroscopy for the placement of distal interlock screws.

The purpose of our study was to describe our technique using the SIGN nail for knee arthrodesis and assess the clinical outcomes. We hypothesized that this technique and construct would be safe and clinically effective for knee fusion.

## Methods

**Figure 1.**
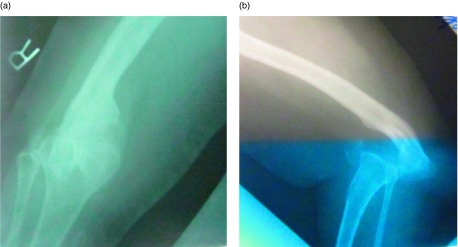
Preoperative anteroposterior (a) and lateral (b) radiographs of congenital knee dislocation.

**Figure 2.**
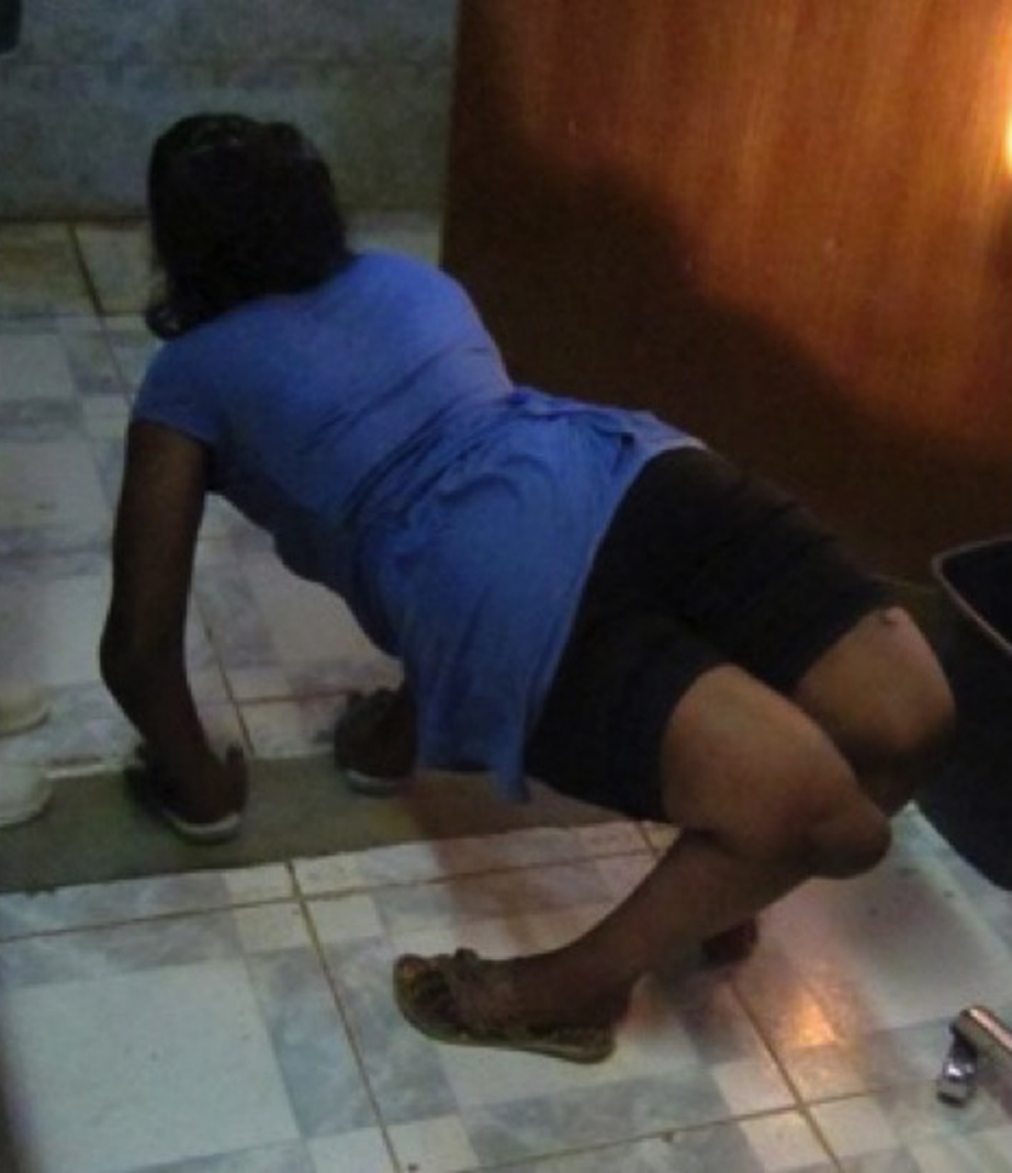
Preoperative photograph of patient with congenital knee dislocations.

This retrospective study approved by our institutional review board included all patients undergoing knee arthrodesis with a SIGN nail (SIGN Fracture Care International, Richland, Washington) at our institution [3]. In all, six consecutive patients (three males, seven knees) with an average age of 30.5 years (range, 18–50 years) underwent a fusion by the described method (Table 1). Preoperative diagnoses included knee tuberculosis in two patients, congenital knee dislocation in two knees (one patient) (Figures 1 and 2), end-stage bacterial septic arthritis in one knee, malunited spontaneous fusion in one knee, and a 90° flexion contracture in one knee with severe gout.

**Table 1.** Patient demographics, diagnoses, and clinical outcomes.

Patient	Age years	Sex	Diagnosis	Followup	Complications	Ambulation aide
1	18	Female	Resolved infection; 90° spontaneous fusion	12 months	None	None
2	24	Female	Treated TB	14 months	None	None
3	42	Male	Severe gout; 90° flexion contracture	12 months	None	Cane; contralateral knee gouty arthritis
4	50	Male	Resolved infection	12 months	None	None
5	25	Male	Active infection	9 months	None	None
6	27	Female	Bilateral congenital knee dislocations	8 months right knee	None	Crutches
				8 months left knee	none	Crutches
Minimum	18			8		
Maximum	50			14		
Average	30.4			10.7		
Standard Deviation	11.3			2.3		

### Surgical technique

Our surgical technique utilized a straight anterior incision distal to the tibial tubercle and extending proximally to the distal third of the thigh; a medial parapatellar approach was then performed. Free-hand bone cuts with an oscillating saw were made perpendicular to the mechanical axis and then adjusted to obtain neutral alignment of the limb. Occasionally the collateral ligaments were sacrificed to permit full knee extension but were maintained when able to contribute to compression at the arthrodesis site. In several knees with severe flexion contractures, the posterior capsule was released from the femur and/or tibia to obtain extension while limiting the amount of additional bone resected to avoid excessive shortening of the bone and resulting limb-length inequality. The goal was to obtain full extension with minimal effort after the bone cuts were made. Knees were placed in approximately 10 degrees of flexion and neutral coronal alignment. Steimann cross pins can be used to maintain preliminary alignment and rotation during preparation for intramedullary nailing.

**Figure 3.**
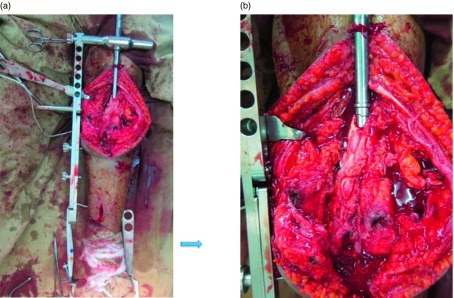
Intraoperative photograph of medial peripatellar exposure and nail entry point in anteromedial femur 10–14 cm above knee (a). Note the expanded cortical window (b) to permit entry of the nail without creating stress riser could cause a fracture when inserting the proximal bend of the “tibial” SIGN nail.

An oblong entrance point approximately 12 cm (10–14 cm depending upon limb length) above the joint line was made on the anterior medial aspect of the distal femur and carefully enlarged with osteotomes and rongeurs (Figure 3). Making this entrance large enough for easy entry and advancement of the tibial nail with a proximal Herzog bend without causing a fracture of the anterior cortex. The canals of the tibia and femur were hand reamed to account for the osteopenic bone that was present in the affected limb as a result of limited weightbearing. The nail was inserted but allowed to sit slightly proud proximally to allow cortical contact on both sides of the nail for better fixation.

The nail was then locked distally. SIGN intramedullary nails do not require fluoroscopy for the placement of distal interlock screws as the instrumentation includes a target arm extension that will give the exact distance of the interlock hole down the nail. The interlock screw is designed to be wider on the near cortical drill hole than the far cortical hole, permitting over-drilling of the near cortex and insertion of a cannulated slot finder/drill guide for drilling of the far cortex in a nail that may be slightly bent from insertion. In cases where knee extension was easily obtained, the knee surfaces were compressed by backslapping the nail after the distal interlock screw was placed.

Finally, the nail was proximally locked through the proximal locking guide arm. The parapatellar arthrotomy and skin were closed in standard fashion. The patella was frequently removed for skin closure while leaving the extensor soft-tissue complex intact.

In two knees with active infection, the joint was debrided, bone cuts were made, and then an external fixator was applied with the knee in extension for one to three months. In one case with a bacterial infection and a 90° flexion contracture, tibial skeletal traction was applied until full extension was obtained (3 weeks) at which time an external fixator was applied to hold the knee in extension. External fixators were left in place while antibacterial/antituberculosis medications were administered. When clinical and laboratory findings indicated the infection was controlled, the external fixator was removed and the nail was inserted using the described technique.

### Postoperative protocol

Knee immobilizers were used for the first six weeks when there was poor bone quality and/or questionable fixation. Weightbearing as tolerated with crutches was immediately permitted in all cases.

### Outcomes

Pre- and postoperative physical exam and radiographs were obtained. Clinical and radiographic evidence of fusion was noted as prospectively collected data and reviewed retrospectively. Whether the patients were able to ambulate with or without aide routinely was additionally noted. Final anteroposterior and lateral radiographs of the leg demonstrating intact hardware (unbroken) and fusion were obtained for each knee. Resolution of pain by patient report was noted as clinical evidence of fusion; remission of radiolucent lines and evidence of trabeculae traversing from the femur to the tibia were used to determine presence of radiographic fusion [4]. Additionally, a physical exam to assess ambulation, stability/fusion, lower extremity neurovascular and motor exam, and wound healing were performed at final followup.

## Results

**Figure 4.**
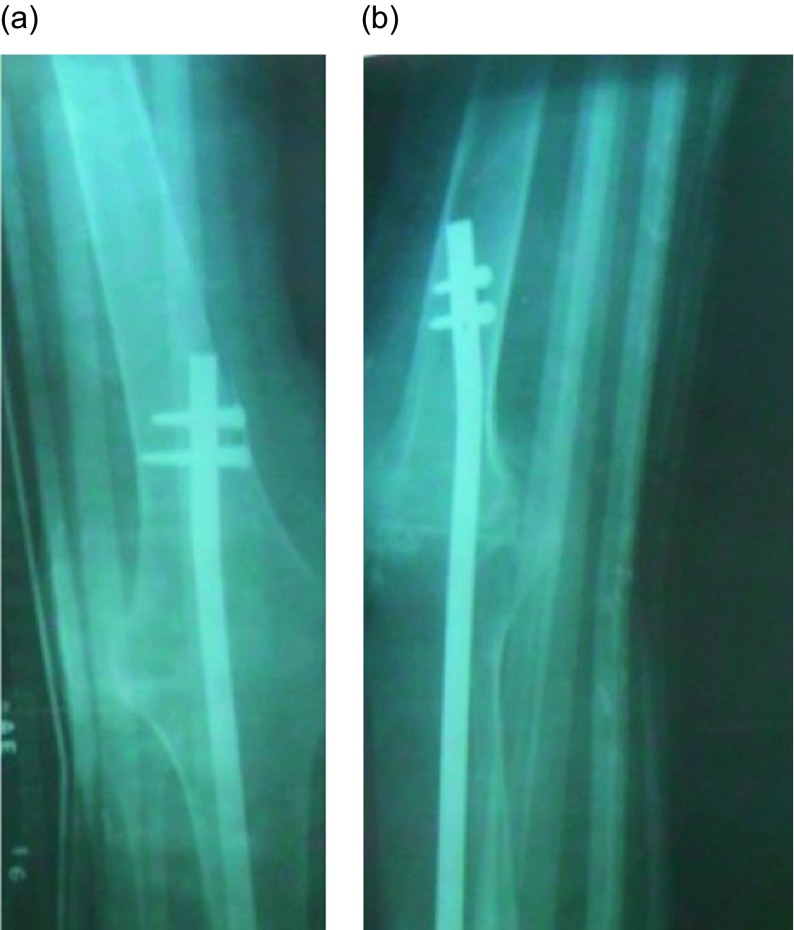
Postoperative anteroposterior (a) and lateral (b) radiographs of knee fusion with SIGN nail construct.

All seven knees had clinical and radiographic evidence of fusion at latest followup; time to clinical and radiographic fusion ranged from three to six months. (Figure 4; Table 1) Two of the patients had temporary (one and three months) external fixators applied after debridement before SIGN nail placement due to active infection (one each with tuberculosis and bacterial infection).

### Outcomes

**Figure 5.**
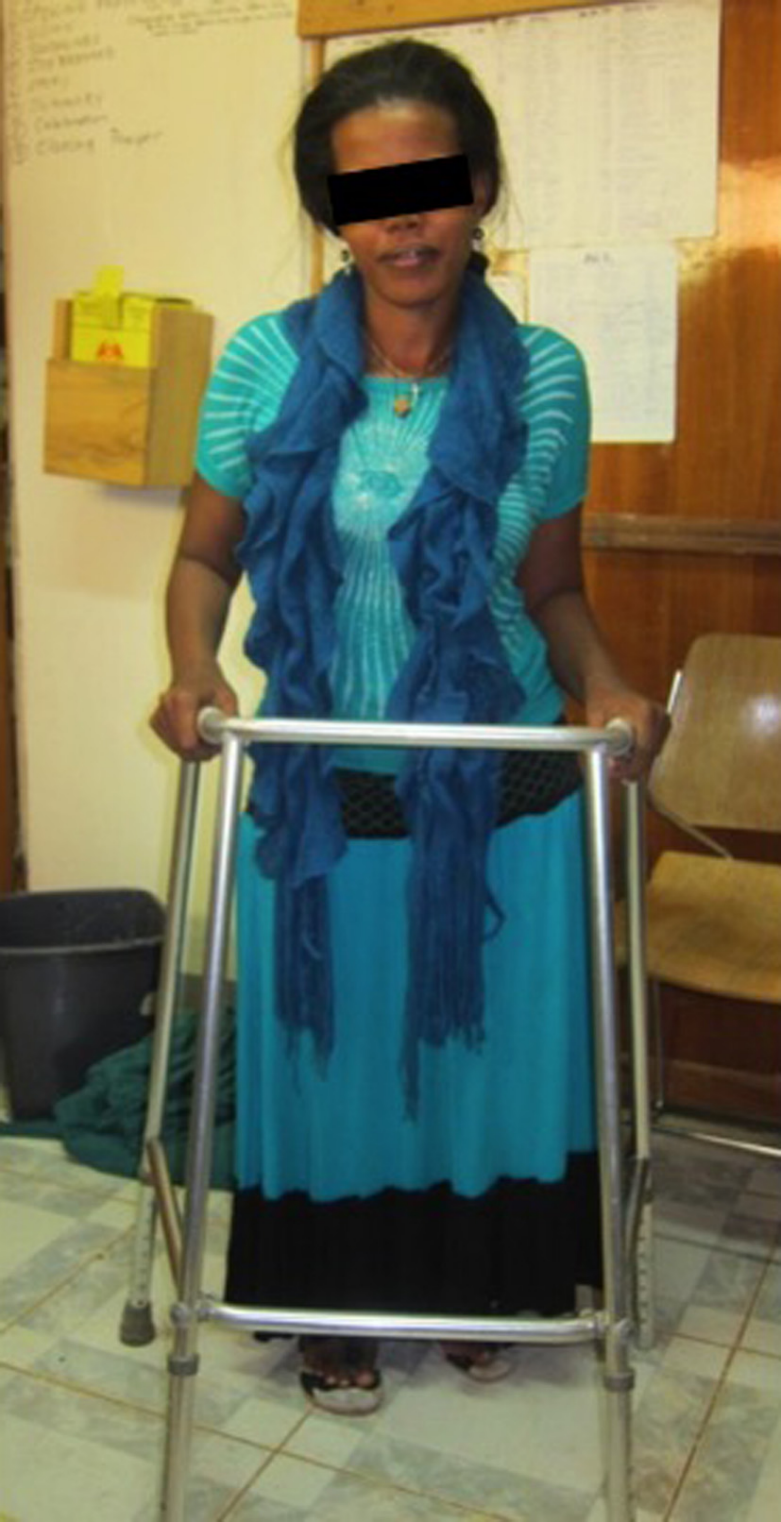
Postoperative photograph of patient with knee fusion for congenital knee dislocations walking with aides.

Four of the six patients were able to ambulate without an aide; one patient requiring an aide had bilateral knee fusions for congenital knee dislocations and the other patient had severe gouty arthritis of his contralateral knee (Figure 5). Final followup averaged 10.7 months followup (range 8–14). All patients reported improved overall physical function and ADLs.

### Complications

There were no postoperative infections, no postoperative neurologic deficits, and no nonunions in our series. No further surgical interventions were necessary after the fusion procedure with SIGN nail insertion.

## Discussion

In the 19th and early 20th centuries, knee arthrodesis was performed in patients with instability secondary to poliomyelitis, articular tuberculosis, septic arthritis/osteomyelitis, irreparable extensor mechanism, and posttraumatic arthritis [1]. The indications for knee arthrodesis in developed countries have changed dramatically over the last century. Arthoplasty options have made arthrodesis a less common procedure though it remains a valuable treatment option for irreparable extensor mechanism and failed two-stage revision attempts [4, 5]. However, the indications for knee arthrodesis in developing countries remain relatively unchanged as polio and tuberculosis have not been eradicated and due to the limited access to arthroplasty. The patients in our current series had severe contractures, congenital dislocations, or severe knee infection.

Construct options for knee arthrodesis include external fixators, intramedullary nails, plate fixation, or a combination of implants. External fixation constructs include monoplanar fixators, biplanar fixators, and circular frames. The advantages of external fixation include minimal soft tissue [6] dissection, decreased blood loss, and the ability to adjust alignment of the limb over time. Additionally, there is no risk of the hardware serving as a nidus for persistent infection since external fixators are removed after fusion. Disadvantages of external fixation include pin tract infections, restricted weightbearing, and lower fusion rates [7]. A distinct advantage of using Ilizarov frames is the ability to perform arthrodesis in the presence of active infection while providing persistent compression and antibiotics with good fusion rates [6].

Advantages of plating include the ability to use the same incision for debridement and fixation. Disadvantages include necessary restrictions on weightbearing as it is not a load-sharing device. Additionally, plates can be prominent in an area with poor soft-tissue coverage risking wound breakdown and often requiring removal of hardware after fusion obtained [1]. While poor bone quality is a concern when performing an arthrodesis with a plate construct, dual plating with locked compression plates has been reported to achieve fusion without implant complications or failures [8].

Nails are reported to have the lowest rates of major complications, and have shorter times to fusion and the highest fusion rates when compared to other implants [2]. The intramedullary nail is a load-sharing implant, which allows early weightbearing and low prominence (a benefit with poor soft-tissue envelope). The long intramedullary nail is inserted antegrade through the piriformis fossa and across the knee joint, providing excellent stability across the long-lever arm of the knee joint. The technique has been reported to have excellent union rates up to 100%, but is technically challenging and violates the hip abductors and can be associated with high rates of reoperation and persistent infection [5, 9, 10]. Vander Griend and Stiehl both reported 100% fusion using a long nail in their separate series of 6 and 4 patients with fusion occurring between 3 and 8 months [11, 12].

In a case series of nine knees with Charcot neuroarthropathy treated with a Kuntscher rod, Drennan reported 100% fusion despite deep infection in 3 [13]. Donley et al. reported fusion in 9 of 11 patients (4 instability, 2 giant cell tumor, 2 failed arthrodesis, 3 fracture nonunion) using a Kuntscher nail with the two nonunions being related to deep infection [14]. In the largest series, Puranen reported 100% fusion in 18 patients within 6 months after arthrodesis using a long intramedullary nail (5 failed prior arthrodesis, 5 instability, 2 tuberculosis infection, 3 osteomyelitis, 3 post-traumatic osteoarthritis) [10]. The patients with infection preoperatively resolved the infection without additional surgery, but all patients continued to ambulate with assistive devices. Reported complications in this series included one patient requiring bone grafting to obtain union by six months and one patient had a broken nail that was exchanged for a larger nail.

More recently, modular nails have been developed to ease nail insertion through the knee joint in each direction; however, these implants are more expensive and not always available in more austere environments [2].

Similar to previous studies, our case series using the SIGN nail achieved 100% fusion without any complications to date. Additionally, four of six patients are able to ambulate without assistive device. Availability and familiarity are additional benefits of using the SIGN nail for knee arthrodesis in developing countries [3]. The SIGN nail is currently available in over 50 countries and many surgeons providing fracture care are already familiar with the implant. Finally, the SIGN nail instrumentation is designed to enable proximal and distal interlock screw insertion without fluoroscopy, an additional benefit in austere environments.

### Limitations

This is a small series of patients with limited followup; however, each patient was followed to clinical and radiographic evidence of knee fusion without breakage of hardware or other complication. Although we report subjectively improved outcomes in all patients, we did not use standardized outcome measures in this study.

## Conclusions

In our small series with short-term followup, we found that our described technique using the SIGN nail for knee fusion is safe and clinically effective and does not require restriction in weightbearing or require fluoroscopy guidance for interlock screw placement.

## Conflict of interest

No financial assistance or external funding was received for this study. Each of the other authors certifies that he has no commercial associations (e.g., consultancies, stock ownership, equity interest, patent/licensing arrangements, etc.) that might pose a conflict of interest in connection with the submitted article.
